# Efficacy and Safety of Pirfenidone in Patients with Progressive Pulmonary Fibrosis: A Retrospective Single-Center Study

**DOI:** 10.3390/life16010011

**Published:** 2025-12-21

**Authors:** Ju Hyun Oh, Jin Han Park, Ji Hoon Jang, Minyoung Her, Een Young Cho, Jae Ha Lee

**Affiliations:** 1 Department of Pulmonary and Critical Care Medicine, Ajou University Medical Center, Ajou University School of Medicine, Suwon 16499, Republic of Korea; angle5018@naver.com; 2 Division of Pulmonology, Department of Internal Medicine, Inje University Haeundae Paik Hospital, Inje University College of Medicine, Busan 48108, Republic of Korea; 1982han@hanmail.net (J.H.P.); saturn80396@gmail.com (J.H.J.); 3 Division of Rheumatology, Department of Internal Medicine, Inje University Haeundae Paik Hospital, Inje University College of Medicine, Busan 48108, Republic of Korea; alsdud92@gmail.com; 4 Division of Radiology, Department of Internal Medicine, Inje University Haeundae Paik Hospital, Inje University College of Medicine, Busan 48108, Republic of Korea; seirosera@naver.com

**Keywords:** interstitial lung disease, treatment, lung function, pirfenidone, mortality

## Abstract

Progressive pulmonary fibrosis (PPF) is an emerging subset of fibrotic interstitial lung diseases (ILD), defined by progressive fibrosis despite standard treatment in patients with other than idiopathic pulmonary fibrosis. The international guidelines recommended the use of nintedanib for PPF, while evidence supporting pirfenidone remains insufficient. In this study, we aimed to evaluate the efficacy and safety of pirfenidone in treating PPF. In this retrospective single-center study, we analyzed clinical data from patients with PPF who were treated with pirfenidone. Lung function data from six months before and after pirfenidone treatment were collected to assess changes over time. Missing values were imputed using a general linear mixed model (GLMM) for longitudinal data analysis. Of 33 subjects, the median age was 65.0 years, and 51.5% were female. Rheumatoid arthritis-related ILD was the most common subtype (45.5%). The median daily dose of pirfenidone was 600 mg, with a median treatment duration of 7.3 months. GLMM analysis showed a significant forced vital capacity (FVC) improvement, from −114 mL in the 6 months before treatment to +47.3 mL in the 6 months after treatment (*p* = 0.001). All adverse events related to pirfenidone were mild. In conclusion, the use of pirfenidone in PPF can potentially reduce the rate of FVC decline in real clinical practice.

## 1. Introduction

Interstitial lung disease (ILD) encompasses a diverse group of pulmonary disorders characterized by inflammation and fibrosis of the interstitium [1,2,3]. Among these, a subset of patients with fibrotic ILD other than idiopathic pulmonary fibrosis (IPF) experience progression of fibrosis despite standard treatment, a condition defined as progressive pulmonary fibrosis (PPF) [1,2,4]. The 2022 ATS/ERS/JRS/ALAT international guideline established diagnostic criteria for PPF: PPF typically manifests as a measurable decline in lung function (e.g., ≥5–10% relative FVC decline within 1 year), worsening respiratory symptoms, and radiologic progression despite appropriate management [1]. Worldwide, PPF has been reported in approximately 20–40% of patients with fibrotic ILD, depending on the distribution of underlying ILD subtypes [5,6]. Prognostically, PPF is strongly associated with poorer survival and more rapid physiological deterioration [5,7,8]. Khor et al. showed that patients with PPF had transplant-free survival comparable to patients with IPF [8].

For IPF, two antifibrotic agents, nintedanib and pirfenidone, have been recognized as standard therapies demonstrating efficacy in reducing lung function decline, lowering the risk of acute exacerbation, and providing survival benefit [1]. Unlike IPF, the only approved treatment for PPF in the current international guidelines is nintedanib, while evidence supporting pirfenidone remains insufficient [2,9]. Nevertheless, because PPF shares similar pathogenesis and disease behaviour with IPF, pirfenidone has been hypothesized to exert similar therapeutic effects in PPF, which have been supported by several studies in progressive fibrotic ILDs [10,11,12]. In the RELIEF trial, pirfenidone attenuated the decline in FVC over 48 weeks in patients with progressive fibrotic ILDs (−36.6 mL in the pirfenidone group vs. −114.4 mL in the placebo group) [11]. Although the study was terminated early due to slow recruitment and limited statistical power, the results remained clinically meaningful, suggesting a potential therapeutic benefit of pirfenidone [11]. Accordingly, the guideline emphasized the need for further studies to confirm the efficacy of pirfenidone in PPF [1,2].

At the time when this study was conducted, no antifibrotic agent was reimbursed for PPF, whereas pirfenidone was approved and reimbursed only for IPF. Consequently, pirfenidone was more readily accessible and was frequently prescribed off-label to patients with PPF who exhibited progressive fibrosis despite conventional treatment. This real-world context, in which antifibrotic therapy was feasible but evidence remained limited, necessitated an evaluation of the efficacy and safety of pirfenidone in patients with PPF.

Therefore, the objective of this study was to evaluate the efficacy and safety of pirfenidone in patients with PPF by analyzing changes in pulmonary function before and after treatment initiation and by assessing treatment-related adverse events and prognosis in real clinical practice.

## 2. Materials and Methods

### 2.1. Study Population

This retrospective single-center study included patients with PPF who received pirfenidone between August 2022 and July 2024 at Inje University Haeundae Paik Hospital in Busan, South Korea. PPF was diagnosed according to the international guideline criteria for non-IPF ILD, which define disease progression as meeting at least two of the following within the past year despite appropriate management: (1) worsening respiratory symptoms such as increased dyspnea or cough, (2) physiological evidence of progression, including an absolute decline in forced vital capacity (FVC) of ≥5% predicted or diffusing capacity of the lungs for carbon monoxide (DLco) of ≥10% predicted within 1 year, or (3) radiological progression on high resolution computed tomography (HRCT), including increased fibrosis, worsening traction bronchiectasis, or emergence of new reticular or honeycombing features [1]. Patients were eligible if they had received pirfenidone treatment for at least 3 months, and their diagnosis and longitudinal management were confirmed through multidisciplinary discussion that included pulmonologists, radiologists, and rheumatologists. The study protocol was approved by the institutional review board of Inje University Haeundae Paik Hospital (IRB 2025-07-016), and the requirement for informed consent was waived due to the retrospective nature of the study.

### 2.2. Clinical Data

Demographic and clinical characteristics were obtained from electronic medical records, including age, sex, smoking history, body mass index (BMI), and ILD subtypes. Information regarding pirfenidone use, including dosage, treatment duration, and concomitant use with corticosteroids or immunosuppressants, was also collected. Spirometry parameters and diffusing capacity of the lung for carbon monoxide (DLco) were measured according to the ATS/ERJ recommendations and expressed as percentages of predicted values [13,14]. The six-minute walk test (6MWT) was performed in accordance with the guideline [15].

### 2.3. Efficacy Evaluation

The primary efficacy endpoints were changes in lung function, including FVC (% predicted and mL) and DLco (% predicted), as well as six-minute walk distance (6MWD), measured from 6 months before to 6 months after pirfenidone initiation. To assess treatment effects over time, pulmonary function and 6MWT results were collected at three predefined time points: 6 months before treatment (pre-treatment period), at treatment initiation (baseline), and 6 months after treatment (post-treatment period). Clinical outcomes, such as acute exacerbation, mortality, and treatment-related adverse events, were also recorded during the follow-up period.

### 2.4. Pirfenidone Administration

Pirfenidone dosing was individualized at the discretion of the physician. Most patients initiated pirfenidone therapy orally at 600 mg/day (200 mg three times daily). In real-world practice, many patients remained at 600 mg/day regardless of tolerability due to the financial burden associated with non-reimbursed pirfenidone in Korea. Dose escalation to 1200–1800 mg/day was attempted when patients tolerated the medication and had no cost-related limitations, typically at 2–4-week intervals. Patients were instructed to take pirfenidone with meals to minimize gastrointestinal adverse effects, and dose adjustments or temporary interruptions were implemented when necessary.

### 2.5. Statistical Analysis

Variables were summarized by frequency and percentage for categorical data and median (IQR, interquartile range) for numeric data. Differences between two time-points were compared with paired ^t^ test or Wilcoxon’s signed-rank test for numeric variables as appropriate. To check if its distribution is normal, we used Shapiro–Wilk test. Group differences were tested using the chi-squared test or Fisher’s exact for categorical data. Data were observed at three different timepoints: 6 months before baseline, baseline, and 6 months after baseline. By the nature of longitudinal data, general linear mixed model (GLMM) was used in the analysis of unbalanced repeated measures designs. A mixed model had random patient intercept and slope terms as well as fixed effect for time. Imputation of missing values was conducted using these mixed-model predictions. Cox regression analysis was performed to identify risk factors for mortality. All statistical analyses were carried out using SPSS 29.0 statistical software (IBM Corp. Released 2023. IBM SPSS Statistics for Windows, Version 29.0.2.0 Armonk, NY, USA: IBM Corp). *p*-values less than 0.05 was considered statistically significant.

## 3. Results

### 3.1. Baseline Characteristics and Treatment

A total of 33 patients with PPF were included (Table 1). The median age was 65 years, and approximately half were male and ever-smokers. About half of the patients (51.5%) were diagnosed with PPF based on both symptom worsening and lung function decline. RA-ILD was the predominant ILD subtype, and most patients demonstrated a UIP pattern on HRCT. Baseline lung function reflected moderate physiologic impairment characteristic of PPF, with a median FVC of 70.0% predicted, a median DLco of 59.0% predicted, and 30.3% of patients requiring home oxygen therapy.

Treatment details are summarized in Table 2. At the time of pirfenidone initiation, most patients were receiving corticosteroids and/or immunosuppressants, whereas 12.1% were not receiving any concomitant treatment. Concomitant therapy remained unchanged throughout the 6-month follow-up period. The majority were treated with reduced-dose pirfenidone, with a median daily dose of 600 mg, and the median duration of therapy was 7.4 months.

### 3.2. Functional Changes After Pirfenidone Treatment

**Figure 1:**
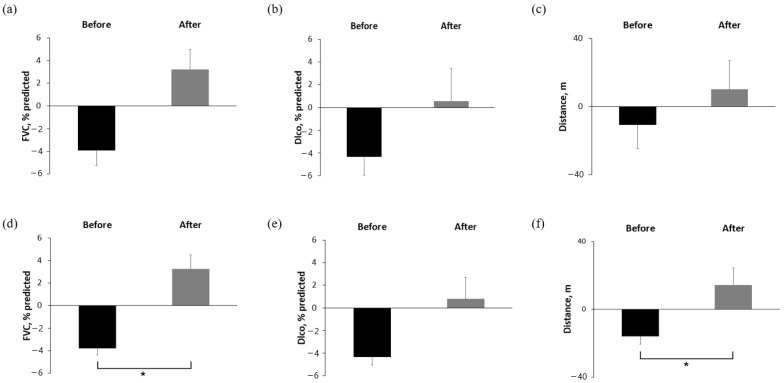
Comparison of absolute change in FVC, DLco, and 6MWD over 6 months before and after pirfenidone administration (**a**–**c**) raw data and (**d**–**f**) missing value imputation with GLMM analysis. * Asterisks indicate statistically significant differences (*p* < 0.05) between two groups, FVC, forced vital capacity; DLCO, diffusing capacity of the lungs for carbon monoxide; 6MWD, six-minute walking distance; GLMM, general linear mixed model.

In the raw data analysis, the mean change in FVC % predicted was −3.93% in the 6 months before treatment and +3.22% in the 6 months after treatment, which was not statistically significant (*p* = 0.174) (Figure 1a). DLco and 6MWD also showed no significant differences in the raw data (Figure 1b,c). However, the GLMM analysis demonstrated a significant improvement in FVC % predicted, from −3.79% predicted in the 6 months before treatment to +3.27% predicted in the 6 months after treatment (*p* = 0.001) (Figure 1d). DLco showed a trend toward improvement (−4.34 vs. +0.79% predicted; *p* = 0.062), while 6MWD significantly improved (−15.9 vs. +14.2 m; *p* = 0.024).

**Figure 2:**
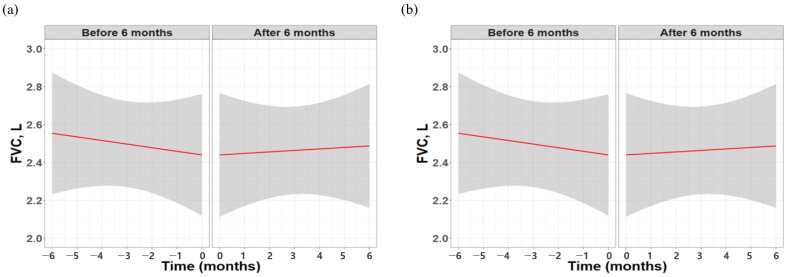
Longitudinal changes in FVC over 6 months before and after initiation of pirfenidone therapy. (**a**) raw data and (**b**) missing value imputation with GLMM analysis. Red lines indicate the modeled trajectory for each period, with shaded areas representing 95% confidence intervals.; FVC, forced vital capacity; GLMM, general linear mixed model.

Additionally, while the raw data showed no significant absolute change in FVC, the results based on the GLMM analysis revealed a marked improvement in trajectory, shifting from −114 mL (95% confidence interval [CI] −172.8 to −55.6 mL) in the 6 months before treatment to +47.3 mL (95% CI −39.2 to −133.8 mL) in the 6 months after treatment (*p* = 0.001) (Figure 2).

### 3.3. Adverse Events During Treatment

Although most patients received pirfenidone in combination with corticosteroids, immunosuppressants, or both, treatment-related toxicities were generally mild. After initiation of antifibrotic therapy, adverse events included anorexia in 21.2% (*n* = 7), photosensitivity in 6.1% (*n* = 2), and pruritus in 15.1% (*n* = 5). Notably, no severe adverse events requiring drug discontinuation were observed.

### 3.4. Prognosis

During a median follow-up period of 23.2 months (IQR, 10.7–58.9), 4 patients (12.1%) died. Causes of death included acute exacerbation (*n* = 1), chronic respiratory failure (*n* = 1), and other causes (*n* = 2). In addition, 6 patients (18.2%) experienced acute exacerbation during the follow-up period. In the univariate Cox regression analysis, a history of acute exacerbation showed the highest risk, with a hazard ratio of 26.00 (95% CI 2.01–336.11; *p* = 0.013) (Table 3).

## 4. Discussion

In this study, we evaluated the efficacy and safety of pirfenidone in patients with PPF at a single center in Korea. Although the analysis of raw data showed only a trend toward improvement in FVC without statistical significance, the results based on the GLMM analysis demonstrated significant improvements in both FVC and 6MWD following pirfenidone treatment. In terms of safety, no severe adverse events or treatment discontinuations were observed.

In the present study, FVC improved by a mean of 47.3 mL over 6 months following pirfenidone initiation in real-world practice. This result is compatible with previous randomized controlled trial findings [10,11]. In a phase 2 study of patients with unclassifiable progressive fibrosing ILD, the decline in FVC measured by site spirometry was attenuated in the pirfenidone group, with a mean change of −17.8 mL over 24 weeks, representing a 95.3 mL advantage compared with placebo (95% CI, 35.9–154.6; *p* = 0.002) [10]. DLco and 6MWD also showed numerical improvements in the pirfenidone group [10]. Similarly, in the RELIEF trial, which enrolled patients with progressive fibrotic ILDs, slope analyses indicated an estimated treatment effect of 3.53% predicted FVC (95% CI, 0.21–6.86), and in terms of absolute volume, the mean change in FVC at week 48 was −36.6 mL in the pirfenidone group compared with −114 mL in the placebo group, favoring pirfenidone [11].

While previous studies have primarily demonstrated an attenuation of lung function decline, the present study showed an improvement in FVC, which should be interpreted cautiously given the small sample size and retrospective design [10,11]. Nevertheless, this finding may be explained by several potential mechanistic factors. Although established fibrotic lesions are generally considered irreversible under current antifibrotic therapies, pirfenidone exerts not only antifibrotic but also anti-inflammatory and antioxidative effects [16,17]. In non-IPF ILDs, fibrotic changes frequently coexist with inflammatory lesions that may represent a reversible component of lung impairment [16,17]. Therefore, these findings may reflect the alleviation of inflammatory responses and improvement in reversible inflammatory components rather than true reversal of fibrosis.

In PPF, the INBUILD randomized controlled trial demonstrated that nintedanib significantly slowed the annual rate of FVC decline compared with placebo (nintedanib: −80.8 mL/year vs. placebo: −187.8 mL/year; between-group difference ≈107 mL/year; *p* < 0.001) [18]. Based on this large randomized controlled trial and other supporting evidence, the current ATS/ERS/JRS/ALAT guidelines recommend nintedanib for patients with PPF who continue to progress despite standard management [1]. In contrast, evidence for pirfenidone in PPF primarily derives from smaller single randomized trials and observational studies, and no head-to-head comparison between the two antifibrotic agents exists [10,11]. Nonetheless, a meta-analysis by Finnerty et al. reported that both agents demonstrate significant antifibrotic effects in progressive fibrosing ILDs [19]. The recent multicenter retrospective study including patients with IPF and PPF showed that combination therapy with nintedanib and pirfenidone improved the rate of FVC decline, reducing the monthly loss from −26.7 mL before combination therapy to −11.1 mL during treatment (*p* < 0.05) [20]. Additionally, the meta-analysis indicated that pirfenidone provided greater reductions in all-cause mortality and serious adverse events in PPF than nintedanib or nerandomilast [21]. Taken together, these findings suggest that pirfenidone may represent a practical and clinically meaningful additional therapeutic option for patients with PPF.

In the present study, a statistically significant improvement in the 6MWD was observed after pirfenidone treatment. Because 6MWD reflects exercise tolerance and is closely associated with health-related quality of life and prognosis in patients with ILD, this finding suggests a potential functional benefit of pirfenidone in patients with PPF [22]. Similar trends have been reported in previous studies. In a systematic review and meta-analysis by Ghazipura et al., pirfenidone-treated patients with PPF demonstrated a mean improvement in 6MWD of 25.2 m compared with placebo (95% CI, 8.3–42.1 m) [12]. However, the absolute magnitude of 6MWD improvement observed in the present study was modest and did not reach the generally suggested minimal clinically important difference of approximately 25–30 m for chronic respiratory diseases and IPF [15,23]. Therefore, although the observed change may indicate a favorable trend in physical performance, its clinical relevance should be interpreted with caution.

In this study, patients with RA-ILD accounted for approximately 45.5% of the ILD population, and 75.8% demonstrated a UIP pattern on HRCT. Although the UIP pattern in non-IPF ILD is generally considered a radiologic feature associated with a higher risk of disease progression and poorer prognosis, an improvement in pulmonary function was observed following pirfenidone treatment in our cohort [24,25]. Previous study has also suggested that pirfenidone may be particularly effective in patients with RA-ILD and those with a UIP pattern [26]. In the TRAIL1 trial of RA-ILD, the annual decline in FVC over 52 weeks was −66 mL in the pirfenidone group compared with −146 mL in the placebo group, indicating a significantly slower rate of decline (*p* = 0.0082) [26]. Moreover, among pirfenidone-treated patients, those with a UIP pattern showed a smaller annual FVC reduction than those without a UIP pattern (−43 mL vs. −85 mL) [26]. Based on this evidence, the 2023 American College of Rheumatology and American College of Chest Physicians guideline for the treatment of systemic autoimmune rheumatic disease–associated ILD conditionally recommended the use of pirfenidone specifically in RA-ILD [27]. Accordingly, our findings suggest that patients with non-IPF ILD exhibiting a UIP pattern should be closely monitored for disease progression, and antifibrotic therapy such as pirfenidone may be considered as part of their management strategy. Notably, this study contributes real-world clinical data from a Korean PPF population with a high prevalence of RA-ILD and substantial low-dose pirfenidone use, adding context that supports and enriches existing randomized evidence.

In this cohort, pirfenidone was administered at a mean daily dose of 600 mg, which is substantially lower than the doses used in previous clinical trial of non-IPF ILDs [10,26]. Nevertheless, treatment efficacy was observed, and the incidence of adverse events was lower than that reported. Korean real-world studies in patients with IPF have also shown that clinical outcomes, including FVC decline and survival, do not differ significantly according to pirfenidone dose, while lower doses are associated with fewer adverse events and better treatment persistence [28,29,30]. These findings suggest that low-dose pirfenidone may represent a practical therapeutic option in real-world settings, particularly when considering patient tolerability and adherence. Furthermore, in Korea, the clinical value of pirfenidone remains significant given the restrictions on nintedanib use related to reimbursement criteria, cost, and tolerability in PPF.

This study has several limitations. First, it was a retrospective, single-center study with a relatively small sample size, which may limit the generalizability of our findings. Second, due to its retrospective nature, unmeasured confounding factors could not be completely excluded. Third, most patients received a median dose of 600 mg/day of pirfenidone, mainly due to cost considerations, and therefore the efficacy of higher doses could not be evaluated in this cohort. Fourth, missing PFT data occurred because some referred patients lacked available or regular PFT assessments, and patients without PFT results could still be included according to the diagnostic criteria for PPF. To reduce potential bias, missing data were addressed using the statistical GLMM approach to account for incomplete observations; however, given that the GLMM-derived estimates are model-based results obtained from a small cohort, they should be interpreted cautiously and considered exploratory findings that supplement the raw paired results. In addition, the very small number of mortality events reduced the stability of the Cox regression estimates and increased the risk of overfitting, making multivariable analysis difficult to apply. Despite these limitations, this study provides preliminary real-world evidence supporting the effectiveness and safety of pirfenidone in Korean patients with PPF.

## 5. Conclusions

In this real-world cohort of patients with PPF, pirfenidone reduced the rate of FVC decline and demonstrated acceptable tolerability, even at reduced doses. These findings suggest that pirfenidone may serve as a valuable therapeutic option for PPF. Further prospective studies with larger cohorts are needed to confirm its efficacy and long-term clinical benefits.

## Figures and Tables

**Table 1:** Baseline characteristics of patients with PPF.

Variables	Numbers
Age, year	65.0 [58.0–71.5]
Male	16 (48.5)
BMI, kg/m^2^	25.1 [23.3–27.7]
Ever-smoker	17 (51.5)
PPF criteria	
Symptom worsening + lung function decline	17 (51.5)
Symptom worsening + Radiologic progression	12 (36.4)
lung function decline + Radiologic progression	1 (3.0)
Symptom worsening + lung function decline + Radiologic progression	3 (9.1)
Classification of ILD	
Rheumatoid arthritis-ILD	15 (45.5)
Sjögren’s syndrome-ILD	6 (18.2)
Systemic sclerosis-ILD	1 (3.0)
Dermatomyositis-ILD	1 (3.0)
Polymyositis-ILD	1 (3.0)
Mixed-connective tissue disease-ILD	1 (3.0)
Microscopic polyarteritis-ILD	2 (6.1)
Cryptogenic organizing pneumonia	1 (3.0)
Idiopathic non-specific interstitial pneumonia	4 (12.1)
Unclassifiable ILD	1 (3.0)
UIP pattern on HRCT	25 (75.8)
Pulmonary function test	
FVC, % predicted	70.0 [51.0–81.5]
DLco, % predicted	59.0 [41.0–68.0]
6-min walk test	
6MWD, meters	441.0 [363.5–504.5]
Baseline SpO_2_, %	97.0 [95.0–98.0]
Nadir SpO_2_, %	90.0 [86.0–94.0]
KL-6, UI/mL	641.2 [435.9–1017.5]
Pro-BNP, pg/mL	69.1 [37.5–156.9]
Home O_2_	10 (30.3)

Data are presented as median [IQR] or number (%) unless otherwise indicated. PPF, progressive pulmonary fibrosis; BMI, body mass index; ILD, interstitial lung disease; UIP, usual interstitial pneumonia; HRCT, high-resolution computed tomography; FVC, forced vital capacity; DLco, diffusing capacity of the lungs for carbon monoxide; 6MWD, six-minute walking distance; SpO_2_, peripheral oxygen saturation; KL-6, Krebs von den Lungen-6; BNP, brain natriuretic peptide.

**Table 2:** Concomitant medications at pirfenidone initiation and treatment details.

Treatments	Numbers
Concomitant treatment at pirfenidone initiation	
None	4 (12.1)
Steroid	12 (36.4)
Immunosuppressants *	4 (12.1)
Steroid + Immunosuppressants *	13 (39.4)
PFD dosage, mg/day	
median [IQR]	600.0 [600.0–900.0]
400	3 (9.1)
600	22 (66.7)
1200	7 (21.2)
1800	1 (3.0)
PFD duration, month	7.4 [3.7–13.1]

Data are presented as median (IQR) or number (%) unless otherwise indicated. PFD, pirfenidone; IQR, interquartile range * included mycophenolate mofetil, azathioprine, cyclophosphamide and rituximab.

**Table 3:** Cox regression analysis for risk factors for mortality.

Variables	HR (95% CI)	*p*-Value
Age	1.25 (1.01–1.56)	0.043
Male	1.07 (0.13–8.67)	0.948
BMI	0.80 (0.58–1.11)	0.177
Ever-smoker	0.93 (0.12–7.55)	0.948
UIP pattern on HRCT	NE ^†^	
FVC, % predicted	0.96 (0.90–1.02)	0.151
DLco, % predicted	0.93 (0.86–1.00)	0.059
6MWD, meters	0.99 (0.98–1.01)	0.357
Baseline SpO_2_	1.24 (0.69–2.23)	0.475
Nadir SpO_2_	0.95 (0.80–1.14)	0.576
Home O_2_	9.43 (0.84–105.79)	0.069
KL-6	1.00 (1.00–1.00)	0.703
Pro-BNP	1.00 (1.00–1.01)	0.086
Acute exacerbation	26.00 (2.01–336.11)	0.013

HR, hazards ratio; CI, confidence interval; BMI, body mass index; UIP, usual interstitial pneumonia; HRCT, high-resolution computed tomography; FVC, forced vital capacity; DLco, diffusing capacity of the lungs for carbon monoxide; 6MWD, six-minute walking distance; SpO_2_, peripheral oxygen saturation; KL-6, Krebs von den Lungen-6; BNP, brain natriuretic peptide; NE, not estimable: ^†^ failed to converge, because all deaths occurred in the UIP group.

## Data Availability

The datasets generated and/or analyzed during the current study are not publicly available due to institutional and ethical restrictions related to patient privacy and cannot be shared.

## Abbreviations

The following abbreviations are used in this manuscript:

DLco	Diffusing capacity of the lungs for carbon monoxide
FVC	Forced vital capacity
GLMM	General linear mixed model
HR	Hazard ratio
HRCT	High-resolution computed tomography
ILD	Interstitial lung disease
IPF	Idiopathic pulmonary fibrosis
KL-6	Krebs von den Lungen-6
PFD	Pirfenidone
PFT	Pulmonary function test
PPF	Progressive pulmonary fibrosis
SpO_2_	Peripheral oxygen saturation
UIP	Usual interstitial pneumonia
